# High prevalence of complementary and alternative medicine use among patients with sickle cell disease in a tertiary hospital in Lagos, South West, Nigeria

**DOI:** 10.1186/s12906-017-1812-2

**Published:** 2017-06-07

**Authors:** A. A. Busari, M. A. Mufutau

**Affiliations:** Department of Pharmacology Therapeutics & Toxicology, College of Medicine, University of Lagos, Idi-Araba, Lagos, Nigeria

**Keywords:** Complementary and alternative medicine, Sickle cell disease, Prevalence, Nigeria

## Abstract

**Background:**

Attention and interest in the use of complementary and alternative medicine (CAM) has been reignited globally, most especially in patients with chronic diseases. Sickle cell disease (SCD) is one of such chronic diseases associated with devastating clinical and psychosocial consequences, thus leading those affected to seek alternative treatment apart from orthodox medicine. Hence, this study aimed to determine the prevalence, pattern and tolerability of the use of CAM in patients with SCD in the Lagos University Teaching Hospital (LUTH).

**Methods:**

This was a cross-sectional survey of 200 respondents with SCD attending the hematology clinics of the Lagos University Teaching Hospital over a period of 3 months. Data on socio-demographic characteristic, clinical profile, the types and sources of CAM used were collected using a well structured pretested questionnaire. The data obtained were analyzed using Statistical Package for Social Sciences (SPSS®) version 17.

**Result:**

Of the 200 patients who participated in the study, 113; 56.5% were males and 87; 43.5% were females. Majority of the SCD patients were 1–10 years old and their mean age was 18.8 ± 14.39 years. CAM was reportedly used by 88.5% of the respondents. Biological (herbal) products 156; 62.9% were the most commonly used CAM, followed by alternative medical systems 52; 20.9% and mind-body interventions 30; 12.1%. Relations, friends and neighbors influenced 85.2% of CAM users by recommending CAM to them. Tolerability of CAM was perceived to be excellent as only 33 (18.6%) of the respondents abandoned the use of CAM. Comparing CAM users and CAM non-users, there was no statistical significant difference in the proportion of those >18 years (45.76% vs 52.17%; *p* = 0.658), those who experienced two or more crises (51.41% vs 34.78%; *p* = 0.183), and those with stable haemoglobin concentration of >7 g/dL (15.81% vs 8.69%; *p* = 0.539) More patients among CAM non-users (91.30%) significantly spend over 3000 Naira (USD 15) per month on medicine than CAM users (4.51%) (*p* < 0.001).

**Conclusion:**

CAM use is highly prevalent among adults with sickle cell diseases in Nigeria. CAM is well tolerated and relatively affordable by these patients. Clinician awareness and understanding of the factors influencing the use of CAM and the potential herbal-orthodox medicine interaction are crucial during hospital treatment of these patients.

## Background

Complementary and alternative medicine (CAM) is any practice that is perceived by its users to have the healing effects of medicine, but does not originate from evidence gathered using the scientific method that is part of biomedicine, or is contradicted by scientific evidence or established science [1, 2].

CAM is classified into five major categories: **A**. *Alternative medical systems* (e.g. traditional oriental medicine, acupuncture, Ayurveda, naturopathy, homeopathy, Native American healing, Tibetan medicine). **B**. *Mind-body interventions* (meditation, hypnosis, dance, art and music therapy, spiritual healing, and prayer). **C**. *Biologic – based therapies* (herbal medicine and dietary supplements, special diets, and orthomolecular medicine). **D**. *Manipulative and body-based methods* (chiropractic, massage, the Feldenkrais method, other “body work” systems, and aspects of osteopathic medicine such as craniosacral work), *and* **E**. * Energy therapies* (reiki, therapeutic touch, and other methods of affecting the “bioelectric field” of the body) [3]. When health care providers and facilities offer both types of care, it is called integrative medicine.

The use of CAM is on the increase globally with a high prevalence in developing countries. About 80% of the population in developing countries is dependent on traditional healing methods, including herbal remedies for health maintenance and therapeutic management of diseases [2]. According to the World Health Organization (WHO), herbal medicines are the first line of treatment for 60% of children with high fever [4]. In Nigeria, Ghana, Mali and Zambia, 60% of children with malaria are also treated with herbal drugs [5]. Herbal medicine is popular among the urban population in Lagos but they appear to be ignorant of its potential toxicities. [6] About 85% of Nigerians are known to use and consult traditional medicine for healthcare, social and psychological benefits and also due to poverty and disillusionment with conventional medical care [2, 7]. Herbal remedies are the most popular component of traditional medical practice in developing countries [4]. The use of herbal remedies is relatively common in most developing countries and has been reported A prevalence of 20–80% for herbal remedy use has been reported in the Caribbean, Trinidad, South Africa and Nigeria [8–10]. The use of herbal remedies for young and old patients with chronic health conditions such as diabetes, asthma, epilepsy, hypertension, HIV infection and cancer has also been widely reported [7, 10, 11].

Sickle cell disease (SCD) is one of such chronic diseases associated with devastating clinical and psychosocial consequences thus leading those affected to seek alternative cure apart from orthodox medicine. One of the hallmarks of this disease is intermittent, unpredictable pain episodes of varying intensities [12]. Pain in SCD presents distinctive challenges for patients, families, and health-care professionals. It is the most frequent problem experienced by people with SCD and has profound effects on comfort and function at work, school, play, and social relationships. The frequent painful episodes usually experienced by sickle cell patients are the most common cause of hospitalizations in these people [12]. In recent time, many SCD patients are beginning to turn to CAM to help manage their painful episodes and other complications [12, 13]. The aim of this survey is to determine the prevalence, pattern and tolerability of the use of CAM in patients with SCD attending clinic in a tertiary hospital in Lagos.

## Methods

### Study design and location

This was a descriptive cross-sectional survey of respondents with sickle cell disease attending the hematology clinics of the Lagos University Teaching Hospital (LUTH) between Julyand October 2015. LUTH is a tertiary health care centre situated in Surulere, Lagos, South-West Nigeria. The hospital was established to provide tertiary care to the inhabitants of Lagos and its environs. It is a major referral centre for chronic medical conditions including sickle cell disease. Two hundred serially attending adults and children respondents with sickle cell disease who gave informed consent were studied using a pre-tested questionnaire. Children who could not give information were interviewed through their parents or guardians.

### Sample size determination

Sample size was calculated using fisher’s formula; N = Z^2^Pq/d^2^ [14]^,^ where N = minimum sample size, Z = Standard deviation set at 1.96 with confidence level of 95%, P = Prevalence at 95% confidence level (set at 36% prevalence of CAM in SCD as obtained from previous study [15]), q = (1-P), and d = level of precision (set at 7.5% margin of error). The calculation yielded a sample size of 157 would be adequate for the study. However, this was increased to 200 so as to make up for any withdrawal from the study..Patients who were critically ill, pregnant and those who failed to give informed consent were excluded. Overall, a total of 200 patients were enrolled for this study using a simple random selection technique.

### Data collection

A well structured pre-tested questionnaire and case records of the respondents were used to collect data. Socio-demographic characteristic and clinical profile of sickle cell disease patients including age, gender, level of education, types of haemoglobinopathy, stable haemoglobin level, types of crises, frequency of crises and treatment given were documented. Data on CAM utilization, pattern, sources and cost of CAM were also gathered. Information on potential side effect and tolerability of CAM were recorded. Clinical details about SCD were extracted from case notes while information about the demographics and CAM use were obtained from the patients. Children were interviewed through their parents.

### Statistical analysis

The data obtained were analyzed using Statistical Package for Social Sciences (SPSS®) version 17. Categorical variables were presented as percentages or proportions while continuous variables were presented as mean ± standard deviation (SD). Fisher’s exact test was used to compare percentages while a non-parametric (Mann-Whitney U) test was used to compare the data for CAM and CAM non-users. A *p*-value of less than 0.05 was taken as statistically significant.

### Ethical consideration

Ethical approval was granted by the Health Research Ethics Committee (HREC) of the LUTH. Participants were adequately informed in a language that they understood about the nature, potential benefits and risk of the study. Written informed consent was obtained from each competent patient, guardian or patient’s next-of-kin. All data collected from the participants were kept confidential.

## Result

Out of the 200 respondents interviewed 113 (56.5%) were males and 87 (43.5%) were females with male-female ratio of 1.3:1. Table 1 shows the socio-demographic pattern and CAM utilization of the respondents. The ages of patients enrolled in the study were between 1 and 49 years. Patients most commonly presenting with SCD were those aged 1–10 years. The mean age was 18.8 ± 14.39 years. Majority of the respondents were Yoruba ethnic group 120 (59.4%), Christians 116 (57.4%) and those with secondary school education and below 150 (70.3%). All of the respondents had at least one episode of crisis per year while 50% had 2 episodes of crises per year. Vaso-occlusive painful crisis was the most prevalent type of sickle cell crisis (81.5%). With regards to the utilization of CAM, 88.5% of the patients indicated using at least one form of CAM.

**Table 1:** Socio-demographic characteristic and frequency of CAM utilization in respondents

Characteristic	Frequency	Percent
Mean Age (SD)	18.79 ± 14.39	
Age		
< 10	92	46
10–20	21	10.5
21–30	23	11.5
31–40	52	26
> 40	12	6
Gender		
Male	113	56.5
Female	87	43.5
Ethnicity		
Hausa	21	10.5
Igbo	56	28.0
Yoruba	120	60.0
Others (Edo)	3	1.5
Religion		
Christian	116	58
Muslim	84	42
Level of education		
No formal education	11	5.5
Secondary and below	150	75.0
Above secondary	39	19.5
Frequency of crises/year		
Once	101	50.5
Twice	99	49.5
Type of crises		
Aplastic	10	5.0
Haemolytic	16	8.0
Priapism	4	2.0
Stroke	7	3.5
Vaso-occlusive bone pain	163	81.5
CAM utilization		
Yes	177	88.5
No	23	11.5

**Fig. 1:**
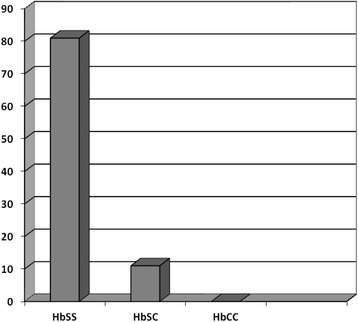
Pattern of Haemoglobinopathies in the respondents with SCD

Figure 1 shows the pattern of Haemoglobinopathy in the respondents with SCD. All the participants had sickle cell anemia (SCA), of which HbSS genotype (178; 89%) was predominant.

**Fig. 2:**
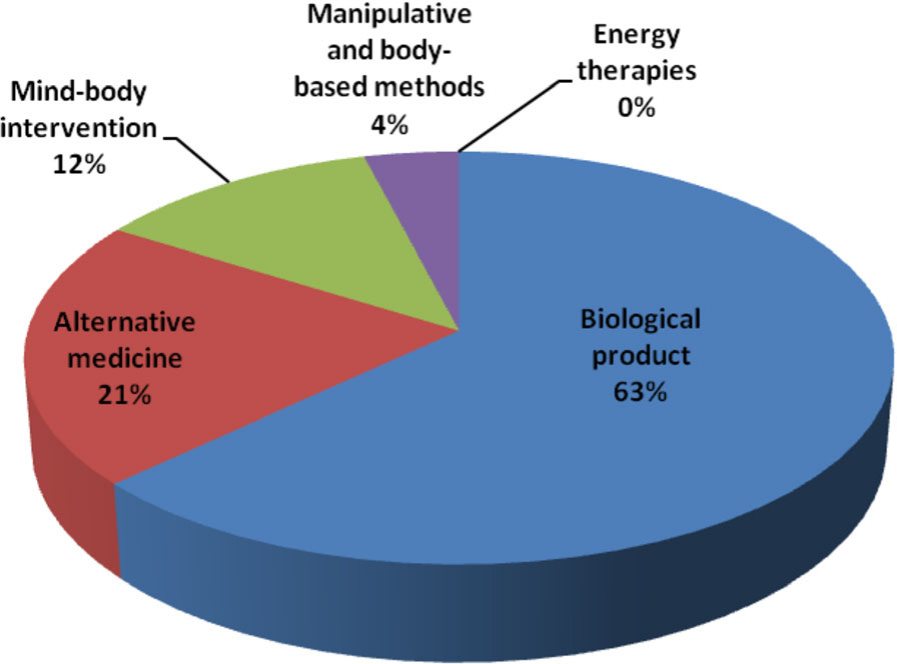
Pattern of categories of CAM utilization among the respondents

In Fig. 2, Shows Pattern of categories of CAM utilization among the respondents Biological products were the most commonly utilized CAM among SCD patients. A total of 156 (62.9%) respondents used biological products. The use of alternative medical systems was reported in 52 (20.9%) of the respondents, while other categories of CAM used were mind-body interventions 30 (12.1%) and manipulative body-based methods 10 (4.0%) respectively.

Table 2 shows pattern of CAM components used by the respondents. Herbal medications constituted the most utilized form of biological products (40;16.1%). Other types of biological agent used included *Aloe vera* (16;6.5%), forever living (25;10.0%), ginger (24;9.7%) and garlic (1;5.2%). The second most prevalent CAM used was alternative medical systems (21%). Thirty (12.1%) respondents believed spiritual healing can change their state of health making mind-body interventions to be the third most prevalent form of CAM used in sickle cell disease patients.

**Table 2:** Pattern of CAM components used by the respondents

Characteristic	Frequency	Percent
Biological product		
Herbal medication	40	16.1
*Aloe vera*	16	6.5
Forever living®	25	10.0
Ginger	24	9.7
Garlic	13	5.2
GNLD®	0	0
Jobelyn®	20	8.1
Tianshi	2	0.8
Lemon grass	18	7.3
Ginseng	1	0.4
Ciklavit®	5	2.0
High/mega dose vitamins	2	0.8
Poly herbal tea	0	0
Special diet and supplements	0	0
Alternative medical systems		
Body scarification	10	4.0
Black soap bath	10	4.0
Blessed/anointed water	14	5.6
Concoction	10	4.0
Chinese medicine	6	2.4
Homeopathy	2	0.8
Ayuveda	0	0
Mind-body interventions		
Spiritual healing/prayer	30	12.1
Meditation	0	0
Divination/incantation	0	0
Manipulative and body-based methods		
Massage	10	4.0

Table 3 shows comparison of the age, frequency of crises, haemoglobin concentration and financial burden for medicines between CAM and CAM non-user. Comparing CAM users and CAM non-users, there was no statistical significant difference in the proportion of those >18 years (45.76% vs 52.17%; *p* = 0.658), those who experienced two or more crises (51.41% vs 34.78%; *p* = 0.183), and those with stable haemoglobin concentration of >7 g/dL (15.81% vs 8.69%; *p* = 0.539) More patients among CAM non-users (91.30%) significantly spend over 3000 Naira (USD 15) per month on medicine than CAM users (4.51%) (*p* < 0.001).

**Table 3:** Comparison of the age, frequency of crises, haemoglobin concentration and financial burden for medicines between CAM and CAM non-users

Characteristics	CAM user (*n* = 177)	CAM non-user (*n* = 23)	*p*-value
Age category >18 (years)	81(45.76%)	12(52.17%)	0.658
Experienced two or more crises	91(51.41%)	8(34.78%)	0.183
Stable Hb conc >7 (g/dl)	28(15.81%)	2(8.69%)	0.539
Cost of Drug >3000/15 (/USD)	8(4.51%)	21(91.30%)	<0.001

*USD* US dollars, *N* Nigeria naira, *Hb* Haemoglobin, *conc* concentration

In Table 4, relationship between demographic characteristics and CAM utilization by the respondents was reported. Being Yoruba ethnic and Christian religious group appeared to be associated factors of CAM utilization. {Yoruba 65.31% vs 30.43%, *p* < 0.001; Christian 64.73% vs 17.39%, *p* < 0.001)}. However, there was no statistical significant difference in gender, level of education, type of Haemoglobinopathy and type of sickle cell crises between CAM and CAM non users among the respondents.

**Table 4:** Relationship between the demographic characteristics and CAM use status of the respondents

Characteristic	CAM user	CAM non-user	*P* value
Gender			
Male	81(46.82%)	6(26.08%)	0.056
Female	96(55.49%)	17(73.91%)	
Ethnicity			
Hausa	8(4.62%)	13(56.52%)	<0.001^a^
Igbo	53(30.63%)	3(13.04%)	
Yoruba	113(65.31%)	7(30.43%)	
Others (Edo)	3(1.73%)	0(0%)	
Religion			
Christian	112(64.73%)	4(17.39%)	<0.001^a^
Muslim	65(37.57%)	19(82.60)	
Level of education			
No formal education	7(4.04%)	-	0.500
Secondary and below	147(84.97%)	18(78.26%)	
Above secondary	23(13.29%)	5(21.73%)	
Haemglobinopathy			
HbSS	156(90.17%)	22(95.65%)	0.245
HbSC	21(12.13%)	1(4.34%)	
Type of crises			
Aplastic	9(50.54%)	1(4.34%)	0.684
Haemolytic	13(7.34%)	3(13.04%)	
Priapism	4(2.25%)	-	
Stroke	7(3.95%)	-	
Vaso-occlusive bone pain	144(81.35%)	19(82.60%)	

^a^ = Statistic significance

The sources of information and perceived benefit of CAM by users is reported in Tables 5 and 6. Relatives, friends and neighbors influenced 151 (84.2%) of the users of CAM. However, relatives appeared to recommend the use of CAM the most to the users (109;61.6%). Other sources of information about CAM included the media: radio and newspaper advertisements accounting for 15.8% of the users. Some benefits of CAM reported by the participants include improvement in general wellbeing, relief of symptoms of the illness, and in management of painful crisis. Perceived relief was said to be good in 56.5% and fair in 25.9% of the respondents. Tolerability of CAM was reported excellent as 162 (91.5%) reported no side effect while 33 (18.6%) of the respondents abandoned the use of CAM with claim of some side effects and cost burden during the course of use. Most of the respondents (134; 75.7%) were willing to recommend CAM to others.

**Table 5:** Sources of Information, perceived benefit and side effects of CAM by users

Characteristic	Frequency	Percent
Source of Information		
Relatives	109	61.6
Friends	20	11.2
Neighbours	22	12.4
Television	0	0.0
Radio	16	9.0
Newspaper	12	6.8
Perceived Effectiveness		
Good	100	56.5
Fair	44	24.9
Poor	33	18.6
Ever abandoned CAM		
Yes	33	18.6
No	144	81.4
Recommendation of CAM to others		
Yes	134	75.7
No	43	24.3
Disclosed use of CAM to doctor		
Yes	0	0
No	177	100
Side Effects		
Always	9	5.1
Frequently	4	2.3
Rarely	2	1.1
Never	162	91.5

**Table 6:** Pattern and components of Biological products used by the respondents

Biological product	Components
Herbal medication®	*Eugenia caryophyllata*, piper guineese, aframomum, melegueta, pterocarpu osun
*Aloe vera*	Essential amino acids- isoleucine, leucine, lysine, methionine, phenylalanine, threonine, valine, tryptophan. Non-essential Amino acid- alanine, arginine, asparagine, cysteine, glutamic acid, glycine, histidine, proline, tyrosine, glutamin aspartic acid anthraquinones, lignins, monosaccharides, saponins, sterols, enzymes, minerals, vitamins, salicylic acid
Forever living®	amino acids, anthraquinones, enzymes, minerals, vitamins, lignins, monosaccharide, polysaccharides, salicylic acid, saponins, and sterols
Ginger	Ginger (*Zingiber officinale Roscoe*),
Garlic	*Allium sativum*, Alliin, s-allylcysteine, methiin
GNLD®	Vitamins, Lipids and sterols
Jobelyn®	Sorghum (Sorghum bicolour Moench) leaves, stalk
Tianshi®	Dried spirulina, Gama lilonelic acid,
Lemon grass	*Cymbopogon citrates*, Z-citral, citral, borneol, estragole, methyleugenol, geranyl acetate, geraniol.beta myrcene, limonene, piperitone,citronellal, carene 2, pinene, farnesol, proximadiol terpiniole, (+)-cymbodiacetal, longifolene-(v4)
Ginseng	*Panas ginseng,* Saponin, phytosterol, carbohydrates, sugar, oleanolic acid, amino acids, peptides, mucilage, resin
Ciklavit®	*Cajanus cajan*
High/mega dose vitamins	Vitamin C, vitamin E, vitamin B
Poly herbal tea	Flavanols, anthocyanin, flavones, catechins,quercetin, theanine, myrecetin, tannins, saponins, glycosides, theaflavins
Special diet and supplements	Vitamin supplements, calcium, omega 3, zinc, magnesium, fresh fruits, vegetables, low fat dairy products, legumes, lean meats, dietary fibre.

## Discussion

Previous studies have suggested that CAM utilization is on the increase globally and is being given recognition by health insurance providers in developed countries [3]. CAM appears to be considered by patients with SCA as the other viable alternative for management of the disease complications. Reports on the prevalence of CAM utilization varies greatly in both developed and developing countries. The figures have ranged from 7% to 83% but the average rate across adult studies has been 31.4% [2, 3, 16].

In this study, we report the prevalence of CAM usage among patients with sickle cell disease attending Lagos University Teaching Hospital, a tertiary health facility in an urban area of the of Lagos metropolis. We also explored some possible patient characteristics determining CAM utilization. The frequency of CAM use in this study (88.5%) is higher than those reported in other studies [3, 12, 15, 17, 18]. This variation could be as a result of what is included under the umbrella of CAM, the nature, cultural values, belief systems, religious underpinnings and practices of our society, as well as the cost and degree of accessibility of conventional medicine. Many Nigerians still utilize traditional medical practices to treat diseases and ailments despite current emphasis on conventional treatment [2, 3, 6, 16–20]. However, this high prevalence rate does not agree with the study carried out in American adults where a low prevalence of (36%) in 2002, and (38.3%) in 2007 were reported respectively [16]. The US rate is lower than the 44%–54% previously reported for chronic childhood illnesses by Sibinga et al. in 2006 but higher than the 12%–23% reported for children with acute or stable health conditions as by Ottolini et al., [13, 21]. Furthermore, in the study by Oshikoya et al. in 2008 where the use of complementary and alternative medicines for children with chronic health conditions in Lagos was evaluated, a high prevalence of CAM use (84%) similar to what we observed in our study was reported among children with chronic diseases. However, the specific prevalence (36%) for children with SCA was substantially lower than ours. One may therefore be tempted to believe that globally, chronic health conditions promote CAM use more than acute illnesses. Unfortunately, this may not be true because of variations in the definition of CAM and of differences in population size between different studies. We found herbal medicine to be the most utilized form of biological agents in this survey. This was similar to the study reported by Amira and Okubadejo [17]. Biological products were the most common category of CAM used by the respondents in our survey followed by the alternative medical system and mind-body intervention respectively. This compares very well to the study by Oshikoya et al. [15]. The African man’s affinity for nature also explains why a significant number of the respondents utilize items gotten from natural sources to maintain their health. The adverse reactions to CAMs reported in this study are few as reported in other studies too [22].

Lower side effects were cited as one of the reasons for the use of CAM by 59% of the respondent in the study by Shafiq et al. [23]. However, most of the respondents were also on regular medications prescribed at the clinic; therefore they may be at risk of drug-herb interactions. The risk of adverse herb-drug interactions is likely to be higher in patients who used more than one biological product [23]. Drug-herb interactions have been reported in patients using both herbal CAMs and prescribed medications [24, 25]. The problems with alternative medicines are lack of standardization of doses, possible drug interactions with conventional medicines and side effects. Some herbs, including garlic, ginkgo, ginseng, and St John’s wort can have a significant influence on concurrently administered drugs [26, 27]. Herbal medicines may mimic, decrease, or increase the action of prescribed drugs [27]. This can be especially important for drugs with narrow therapeutic windows and in sensitive patient populations such as older adults, the chronically ill and those with compromised immune systems [26, 27]. Although many of the respondents in this study claimed to have improvement in health, efficacy of the herbal product taken is not verified. There is the need for more studies to unfold the efficacy, risk and toxicity of these herbal concoctions. In Nigeria, the use of herbal remedies which are perceived to be cheaper may be on the increase [15, 17]. This may be due to the increasing costs of orthodox medicines and the poor economic status of the individual patient [Table 3].

The influence of relatives, friends and neighbors on a patient’s decision to use CAM was also reported. The high percentage (85.2%) of respondents influenced in this study by relatives, friends and neighbors to use CAM is comparable to that previously reported by Lanski et al. [28]. None of the respondents in this study disclosed the use of CAM to their doctors in contrast to the 63% and 66% who disclosed CAM use in other studies [26]. This suggest that clinicians are not likely to know that their patients are adding more drugs than prescribed and unable to predict effectiveness of the drugs they prescribed, understand or interpret follow up report on these drugs. Monitoring drug-drug interactions may also be difficult. It is advisable those clinicians deliberately ask their patients or include it in their follow up routine.

It is interesting to note that many of the respondents were willing to recommend CAM to others. The reasons for this are not readily known from this study. However, possible reasons may include the strong advertisements by alternative practitioners that CAM is a panacea to all diseases thus encouraging patients to try them out. Although this study has shown a very high prevalence of CAM use in sickle cell patients but the relatively small sample size is a limitation. Other limitations of this study include the fact that it was conducted as a single institution survey; a multicentre study may be required in the future in order to give wider coverage of participants. Furthermore, our study did not evaluate direct interaction between CAM and conventional medicine in sickle cell patients.

## Conclusion

CAM use is a common phenomenon amongst patients with sickle cell disease in Lagos University Teaching Hospital. They are considered to be beneficial and well tolerated. However, most patients using CAM did not disclose its use with their doctors. It is therefore recommendable that Health-care providers should carefully examine such use of CAM by their patients as they are often used in conjunction with standard medical therapy. Clinical trials of the biological CAM products should be encouraged to ensure that these products are safe for use”.

## A**bbreviations**

CAM: Complementary and Alternative Medicine
HbCC: Haemoglobin genotype CC
HbSC: Haemoglobin genotype SC
HbSS: Haemoglobin genotype SS
HREC: Health Research Ethics Committee
LUTH: Lagos University Teaching Hospital
SCA: Sickle cell anaemia
SCD: Sickle cell disease
SD: Standard deviation
WHO: World Health Organization
